# Acute Heat Stress Remodels Mitochondrial Ultrastructure and Time-Dependent Transcriptomic Landscapes in Bovine Granulosa Cells

**DOI:** 10.3390/ani16182907

**Published:** 2026-09-15

**Authors:** Zijing Zhang, Jia Min, Zhihao Zhang, Tong Yu, Xian Liu, Xiaoting Zhu, Shijie Lyu, Manru Luan, Qiaoting Shi, Yongzhen Huang, Xingshan Qi, Zhao Zhao, Shenhe Liu, Xiangzhou Yan, Eryao Wang, Xiangnan Wang

**Affiliations:** 1 Institute of Animal Husbandry, Henan Academy of Agricultural Sciences, Huayuan Road 116, Zhengzhou 450002, China; vincezhang163@163.com (Z.Z.); 18591837053@163.com (J.M.); zzh18763091053@163.com (Z.Z.); 15136431521@163.com (X.Z.); sjlyu@outlook.com (S.L.); manruluan0108@163.com (M.L.); sqtsw@126.com (Q.S.); yanxiangzhou@163.com (X.Y.); 2 College of Animal Science and Technology, Henan Agricultural University, Pingan Road 218, Zhengzhou 450046, China; yutong@henau.edu.cn (T.Y.); liushenhe@henau.edu.cn (S.L.); 3 Henan Animal Husbandry Technology Extension Station, Zhengzhou 450008, China; liuxian641@163.com; 4 College of Animal Science and Technology, Northwest A&F University, Xianyang 712100, China; hyzsci@nwafu.edu.cn; 5 Henan Biyang County Livestock Technology Service Center, Zhumadian 463000, China; q13938344182@163.com; 6 Nanyang Yamin Agriculture and Animal Husbandry Co., Ltd., Nanyang 473000, China; 15239012685@163.com

**Keywords:** heat stress, transcriptome, mitochondrial, granulosa cell, bovine

## Abstract

Heat stress is a critical environmental factor that compromises reproductive performance in dairy cattle, primarily through deleterious effects on ovarian follicular cells. Granulosa cells (GCs), which provide essential nutritional and hormonal support for oocyte maturation, are particularly vulnerable to hyperthermic insult. However, the early molecular and subcellular events that precede overt cellular dysfunction remain inadequately characterized. This study aimed to investigate the temporal dynamics of mitochondrial morphological alterations and transcriptomic reprogramming in bovine GCs subjected to acute in vitro heat stress. Bovine GCs were exposed to 43 °C for 10, 20, 30, or 40 min, with untreated cells serving as controls. Heat shock protein expression (HSP90 and HSPA1A) was evaluated by qRT-PCR. Intracellular calcium concentration was measured using the Fluo-3 AM fluorescent probe. Mitochondrial ultrastructure was examined via transmission electron microscopy (TEM). Global transcriptional changes were profiled by RNA sequencing (RNA-seq) at 20 and 40 min post-treatment, followed by bioinformatics analysis. Nine selected differentially expressed genes (DEGs) were validated by qRT-PCR. HSP90 and HSPA1A expression was significantly upregulated at 30 and 40 min of heat exposure (*p* < 0.05). Intracellular Ca^2+^ concentration exhibited a biphasic pattern—markedly elevated at 20 min but significantly reduced at 40 min. TEM revealed progressive mitochondrial swelling and cristae disruption at both time points, with more severe damage at 40 min. RNA-seq identified 185 DEGs at 20 min and 831 DEGs at 40 min, with 285 genes commonly dysregulated across both time points. Functional enrichment analysis demonstrated that the 20-min response was dominated by heat shock protein-related pathways, whereas the 40-min response expanded to encompass apoptosis, inflammation, steroidogenesis, and ciliogenesis pathways. qRT-PCR validation confirmed the reliability of the RNA-seq data. These findings provide a mechanistic framework for understanding heat stress-induced ovarian dysfunction and may inform strategies to preserve fertility in dairy cattle during thermal challenge.

## Simple Summary

Heat stress is a major environmental challenge that reduces fertility in dairy cows, yet the early cellular events triggered by elevated temperature remain poorly understood. To investigate the molecular and structural responses of bovine granulosa cells—the cells that surround and support the developing egg—we exposed them to a high temperature (43 °C) for short periods (10, 20, 30, and 40 min). Observations revealed that heat stress caused swelling of mitochondria, the energy-producing units of the cell, and disrupted calcium balance, with calcium levels rising first, then falling as exposure continued. Using RNA sequencing, we identified 185 genes that changed after 20 min and 831 genes after 40 min of heat exposure, revealing a dramatic time-dependent amplification of the cellular stress response. Many of the affected genes are involved in heat protection, cell survival, inflammation, and hormone production. These findings provide new insights into how heat stress impairs ovarian function at the very early stages and may help develop strategies to protect dairy cow fertility during hot summer months.

## 1. Introduction

When high ambient temperature conditions overwhelm the thermoregulatory responses of the cow body, the body temperature becomes elevated and remains high [1]. HS (heat stress, HS) alters direct and indirect impacts on animal performance, such as growth, reproduction and lactation. Environmentally induced hyperthermia (heat stress) compromises reproductive physiology [2], which has deleterious effects on physiological and performance traits, including oocyte maturation, fertilization and subsequent embryonic development in bovines [3]. Heat stress induces the generation of reactive oxygen species (ROS), endocrine disruption, heat shock system activity, mitochondrial autophagy, and molecular-level alterations, thereby impacting embryonic development. Yin reported that HS (41.5 °C for 24 h) disrupted gap junction (GJ) and transzonal projection (TZP) colocalization in porcine GCs, resulting in impaired GCs and decreased rates of oocyte maturation and survival [4]. Cellular and molecular studies have shown that heat stress compromises oocyte quality and embryonic development, mainly through the induction of mitochondrial dysfunction, oxidative stress, and apoptosis in oocytes and granulosa cells (GCs) or cumulus cells [5,6].

GCs are integral components of the ovaries, where they tend to nourish the developing oocyte inside the follicle and regulate the endocrine signals of reproduction at the ovarian axis [6,7]. GCs are suspected to play important roles in reproductive aging, and apoptosis and steroid hormone secretion are used to determine the fate of follicles and ovarian function [8]. Researchers have reported that heat stress impairs the function of GCs and the developmental competence of oocytes [9]. Lian et al. suggested that heat stress induced GC apoptosis through the BAX/BCL-2 pathway and caused ovarian dysfunction [10]. These changes in GCs lead to changes in the developmental competence of oocytes.

Transcriptomic-level studies involving global RNA sequencing may provide essential biological insights into cellular mechanisms, metabolic level changes, and apoptotic and antioxidant pathways. In our previous study, heat stress (43 °C for 20 min) induced apoptosis and mitochondrial dysfunction. To our knowledge, systematic transcriptomic research on GCs under heat stress (43 °C for 20 or 40 min) is lacking. This work aimed to analyze bovine GCs at the transcriptomic level. This study aims to explore this topic to help better frame the hypotheses and guide our understanding of the study’s significance.

## 2. Materials and Methods

### 2.1. Animal Ethics and Sample Collection

All of the animal experimental protocols and care were approved by the Animal Protection Commission of the Henan Academy of Agricultural Sciences, Zhengzhou, China. Ovaries were collected from Holstein female cattle at a local slaughterhouse. As the ovaries were obtained from a commercial abattoir, detailed information regarding the age, parity, and reproductive status of the donor animals could not be ascertained. The ovaries were placed in sterile saline containing 3% antibiotics at 37 °C and transported to the laboratory within 4 h. The collected ovaries were subsequently washed three times with normal saline at 37 °C.

### 2.2. Cell Culture

First, Follicular fluid was extracted from follicles (2–8 mm), placed in a 15 mL tube and centrifuged at 1500 rpm for 5 min. Then, the cell pellet was resuspended in phosphate-buffered saline (PBS), followed by further centrifugation at 1000 rpm for 5 min, twice. The cell density was measured, and only those samples with a density of at least 5 × 10^5^ cells/mL were inoculated into a 10 cm Petri dish (Corning, Corning, NY, USA), followed by the addition of antibiotics (1% penicillin and streptomycin) and 15% fetal bovine serum (FBS) in Dulbecco’s modified Eagle’s medium (DMEM) (Gibco, Cas: 11965118, Thermo Fisher Scientific Inc. Waltham, MA, USA). Finally, the GCs were cultured for 48 h at 37 °C with 5% CO_2_.

### 2.3. HS Treatment

The GCs were first precultured in medium at 37 °C (the optimal and physiologically relevant temperature for cultured mammalian ovarian cells). HS was induced by exposing the cells to heat (43 °C) for different durations (0, 10, 20, 30, and 40 min). Immediately after the culture, the cells and culture media were collected for subsequent analysis. Two experimental groups were established: 0 min vs. 20 min and 0 min VS 40 min, with 0 min as the comparison group and 20 min and 40 min as the experimental groups. The 20 min vs. 40 min was conducted with 40 min as the experimental group and 20 min as the comparison group.

### 2.4. Detection of Ca^2+^ Levels

Fluo-3 AM (CA1180, Solarbio Biotechnology, Beijing, China) was used to detect intracellular Ca^2+^. The Fluo-3 AM working mixture was added to the cells for incubation at 37 °C for 20 min; 3 volumes of Hank’s balanced salt solution (HBSS) containing 1% FBS were added, and the cells were incubated for an additional 40 min. After being washed with HEPES-buffered saline 3 times and fixed with 5% paraformaldehyde, the cells were incubated with DAPI for 10 min in the dark to stain the nuclei. Then, calcium ion detection was performed under a laser confocal microscope (LSM700; Carl Zeiss AG, Oberkochen, Germany) at an excitation wavelength of 506 nm and an emission wavelength of 526 nm. The fluorescence intensity was analyzed with ImageJ (version 1.54, National Institutes of Health, NIH, Bethesda, MD, USA) software.

### 2.5. Transmission Electron Microscopy (TEM)

The GCs were fixed with 2.5% glutaraldehyde fixative at 4 °C for 4 d, embedded in agarose solution, fixed with osmic acid for 2 h, dehydrated in alcohol, and maintained at 37 °C overnight. The samples were subsequently cut into 50–70 nm slices, which were subsequently placed in a copper mesh box to dry at room temperature overnight. Finally, the samples were observed under an HT7800 transmission electron microscope (Hitachi High-Technologies Corporation, Tokyo, Japan) and photographed for analysis. Mitochondrial morphological damage was evaluated by examining the internal ultrastructure. The following features were defined as clear indicators of damage: swollen cristae, markedly widened cristae spaces, dissolution or even loss of cristae membranes, or the appearance of large vacuolar regions within the mitochondrial matrix. To quantify mitochondrial morphological changes, TEM images were analyzed using ImageJ software (version 1.54, National Institutes of Health, USA). For each group (control, 20 min heat stress, and 40 min heat stress), at least 10 mitochondria were randomly selected from three independent biological replicates. All measurements were performed by two independent researchers who were blinded to the experimental groups.

### 2.6. RNA Extraction

Total RNA was isolated from GCs via a TRIzol total RNA extraction kit (Cat. No. 592, Invitrogen, Carlsbad, CA, USA). The RNA quality was examined via 0.8% agarose gel electrophoresis and spectrophotometry. Whole mRNA-seq libraries were generated by ApexBio Technology LLC. (Shanghai, China) via the Hieff NGS^®^ Ultima Dual-mode mRNA Library Prep Kit^®^ (Cat. No. 12310ES, Yeasen Biotechnology (Shanghai) Co., Ltd., Shanghai, China) following the manufacturer’s recommendations.

### 2.7. qRT–PCR

Total RNA was extracted from the GCs via TRIzol reagent and reverse transcribed into cDNA using HiScript Ill All-in-one RT SuperMix Perfect for qPCR (Cat. No. R333, Vazyme, Nanjing, China). Real-time PCR was performed using the LightCycler^®^96SW1.1 instrument (Roche, Basel, Switzerland) using Taq Pro Universal SYBR qPCR Master Mix (Cat. No. Q712, Vazyme, Nanjing, China), with a total reaction volume of 10 μL, in which the concentration of RNA was 200 ng/μL. The *GAPDH* gene was used for normalization. Relative gene expression was calculated via the 2^−∆∆Ct^ method. The primer sequences are shown in Table 1:

### 2.8. Construction of the cDNA Library

High-quality RNA with a 260/280 absorbance ratio of 1.8–2.2 was used for library construction and sequencing. The library was quality-assessed via an Agilent 2100 Bioanalyzer (Agilent Technologies, Santa Clara, CA, USA). The library was sequenced via the Illumina NovaSeq 6000 sequencing platform (paired-end 150) to generate raw reads (Illumina, San Diego, CA, USA).

### 2.9. RNA-Seq Data Analysis

Raw paired-end FASTQ reads were filtered by TrimGalore (v0.6.10) to discard the adapters and low-quality bases via the Cutadapt tool (v4.4). The obtained clean reads were then aligned to the bovine reference genome (ARS-UCD2.0) via HISAT2 (v2.2.1) [11], followed by reference genome-guided transcriptome assembly and gene expression quantification via StringTie (v2.2.1) [12]. Differentially expressed genes (DEGs) were identified via DEseq2 (v1.42.0) (for samples with replications) [13] or edgeR (v4.0.16) (for samples with no replication), with cutoff values of log2|fold-change| > 1 and *p*-adjust < 0.05. ClusterProfiler (v4.10.0) [14] was used to perform functional enrichment analysis for significant DEGs annotated to potential genes in modules on the basis of Gene Ontology (GO) and Kyoto Encyclopedia of Genes and Genomes (KEGG) pathway categories. Terms with *p* values < 0.05 were considered significant. Gene set enrichment analysis (GSEA) was performed via clusterProfiler with a gene list sorted by log2-fold change.

### 2.10. Protein–Protein Interaction (PPI)

The protein–protein interaction network was constructed based on the STRING database (Search Tool for the Retrieval of Interacting Genes/Proteins, version 12.0, https://string-db.org). Gene symbols or UniProt IDs of the target proteins were submitted to the STRING database, and the appropriate species was selected. The network was built using the ‘full STRING network’ configuration to simultaneously present both direct (physical) and indirect (functional) protein associations. The interaction confidence threshold was set to 0.400 (medium confidence). In the resulting network, nodes represent proteins, edges represent pairwise interactions, and the thickness of each edge reflects the confidence level of the corresponding interaction.

### 2.11. Data Analysis

Statistical analysis was carried out with SPSS 26.0 software (IBM Corp., Armonk, NY, USA) and R software (version 4.2.0). For experiments involving multiple time points or multiple treatment groups, one-way analysis of variance (ANOVA) followed by Tukey’s honestly significant difference (HSD) post hoc test was used to determine the significance of differences among groups. For experiments with only two comparison groups, an unpaired two-tailed Student’s *t*-test was applied. All data were tested for normality using the Shapiro–Wilk test and for homogeneity of variance using Levene’s test prior to parametric analysis. A *p* value < 0.05 was considered statistically significant, *p* < 0.01 was considered highly significant, and *p* < 0.001 was considered extremely significant; *p* > 0.05 was considered not significant (ns). Unless otherwise stated, all experiments were repeated independently at least three times (*n* ≥ 3 biological replicates per group). Graphical representation was performed using GraphPad Prism 9.5 (GraphPad Software, San Diego, CA, USA) and the ggplot2 package in R. All data are presented as the means ± standard deviation (SD).

## 3. Results

### 3.1. Time-Dependent Expression of Heat Shock Proteins in Response to Acute Heat Stress

**Figure 1:**
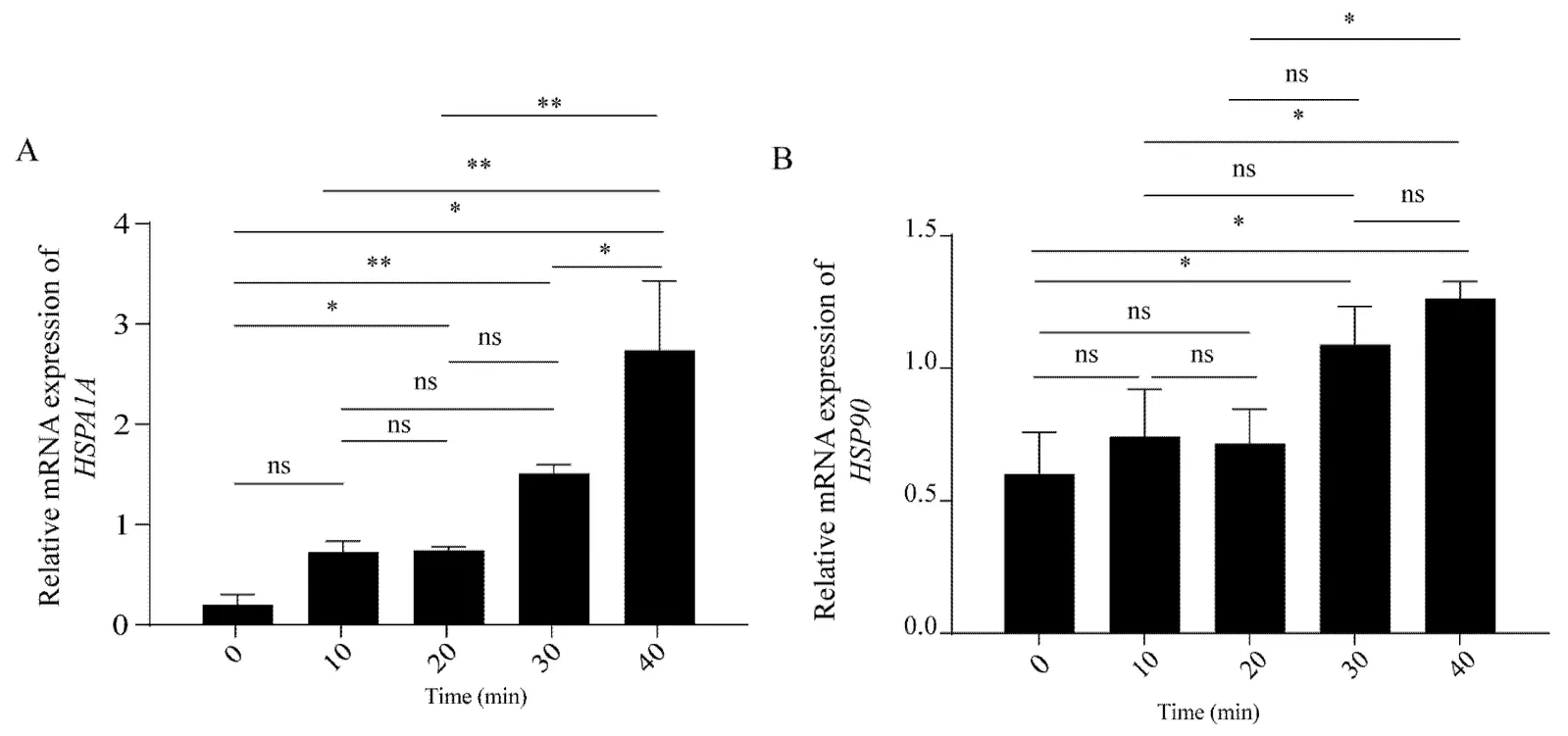
Relative expression of heat stress-related genes. (**A**,**B**) Effects of different durations of heat stress on *HSPA1A* and *HSP90* expression. * *p* < 0.05; ** *p* < 0.01, ns >0.05.

To determine the temporal activation pattern of the heat shock response in bovine granulosa cells (GCs), we exposed cells to 43 °C heat stress for 10, 20, 30, and 40 min and examined the mRNA expression of canonical heat shock genes via qRT-PCR. As shown in Figure 1, *HSPA1A* expression was significantly upregulated as early as 20 min of heat exposure (*p* < 0.05) and continued to increase at 30 and 40 min, indicating a rapid and sustained transcriptional response of this major inducible heat shock protein. In contrast, *HSP90* expression did not reach statistical significance at 20 min but became significantly elevated at 30 and 40 min (*p* < 0.05), suggesting a delayed activation threshold for this constitutively expressed chaperone. These results demonstrate that while GCs mount a rapid heat shock response within 20 min via *HSPA1A*, full engagement of the broader chaperone network, including *HSP90*, requires more prolonged thermal stimulation. Based on these temporal expression profiles and to capture both early and fully established stress responses, we selected 20 min (early response) and 40 min (prolonged response) as the time points for subsequent transcriptomic analysis. Overall, the progressive upregulation of heat shock genes across the treatment duration confirms that acute heat stress exerts a significant negative impact on GCs, triggering a time-dependent activation of cytoprotective machinery.

### 3.2. Concentrations of Ca^2+^ and Mitochondrial Morphology in Granulosa Cells Under Heat Stress

**Figure 2:**
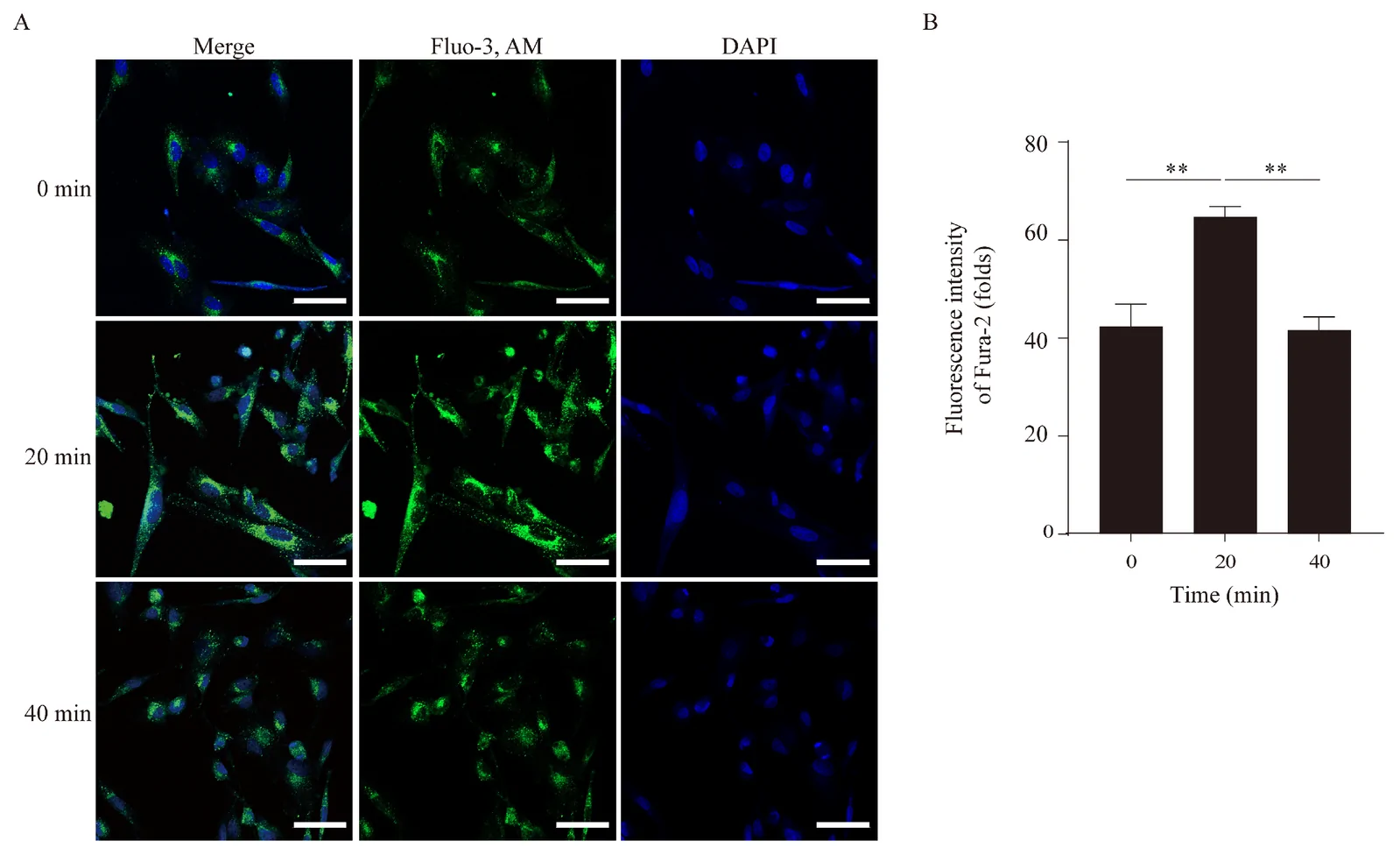
The effect of heat stress on Ca^2+^ concentrations. (**A**,**B**) A laser confocal microscope was used to detect Ca^2+^. scale bar: 50 μm. ** *p* < 0.01.

Mitochondria are the central organelles for steroid hormone biosynthesis. Given their critical role in granulosa cell function, we next examined mitochondrial morphological changes and Ca^2+^ homeostasis following heat stress. The Fluo-3 AM probe results revealed that heat stress significantly increased intracellular Ca^2+^ concentration at 20 min, whereas a significant decrease was observed at 40 min (Figure 2).

**Figure 3:**
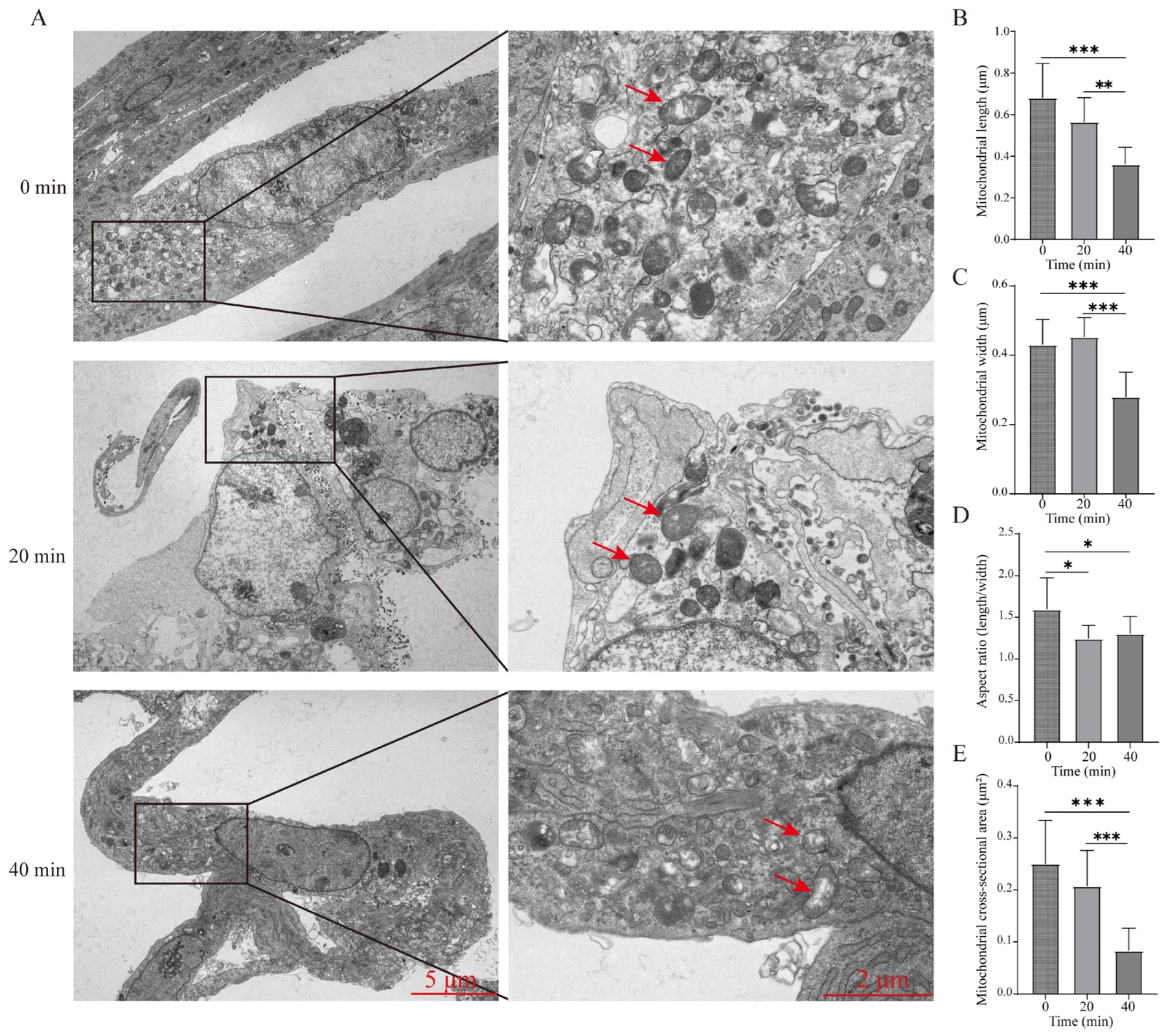
Heat stress-induced mitochondrial morphological damage in bovine granulosa cells. (**A**) Representative transmission electron microscopy (TEM) images showing mitochondrial ultrastructure in the control and heat-stressed groups (43 °C, 20 min and 40 min). Red arrowheads indicate mitochondria. The left panel (scale bar: 5 μm) provides an overview of cellular ultrastructure; the right panel (scale bar: 2 μm) presents higher-magnification views of the boxed regions. (**B**–**E**) Quantitative morphometric analysis of mitochondrial parameters measured using ImageJ software. (**B**) Mitochondrial length (μm), (**C**) mitochondrial width (μm), (**D**) aspect ratio (length/width), and (**E**) mitochondrial cross-sectional area (μm^2^). Data are presented as mean ± SD. At least 10 mitochondria per group were randomly selected from three independent biological replicates for morphometric analysis. * *p* < 0.05, ** *p* < 0.01, *** *p* < 0.001 vs. control group (one-way ANOVA followed by Tukey’s post hoc test).

To further evaluate mitochondrial structural integrity, TEM analysis was performed. Quantitative morphometric analysis using ImageJ software revealed that heat stress (43 °C) induced significant morphological alterations in mitochondria (Figure 3). Specifically, the aspect ratio (length/width) was significantly reduced in both the 20 min and 40 min heat-stressed groups compared to the control group (*p* < 0.05, Figure 3D), indicating a transition from an elongated to a more rounded morphology. Notably, in the 40 min treatment group, mitochondrial length, width, and cross-sectional area were all significantly decreased compared to both the control and 20 min groups (*p* < 0.01 or *p* < 0.001), suggesting progressive mitochondrial contraction or matrix condensation upon prolonged heat exposure.

Collectively, these results demonstrate that heat stress induces significant mitochondrial morphological damage and Ca^2+^ dysregulation, ultimately leading to impaired mitochondrial function in bovine granulosa cells.

### 3.3. Overall Assessment for Mapping Statistics

To ensure the reliability of the transcriptomic analysis, nine cDNA libraries were constructed from bovine granulosa cells subjected to 0 min (control), 20 min, and 40 min of heat stress treatment (43 °C), with three biological replicates per group. As shown in Table 2, high-throughput sequencing generated a total of 47.2 million to 50.3 million raw reads per sample for the control group, 45.8 million to 45.9 million for the 20 min group, and 42.9 million to 49.1 million for the 40 min group. After stringent quality filtering and adapter trimming, clean reads ranging from 41.9 million to 49.2 million were obtained across all samples, with an average of 46.1 million clean reads per library. The clean data per sample ranged from 6.27 Gb to 7.35 Gb, providing sufficient sequencing depth for downstream differential expression analysis. Quality assessment parameters demonstrated the high quality of the sequencing data, with Q20 scores exceeding 98.64% and Q30 scores exceeding 95.95% for all samples. The average GC content was 50.57% (ranging from 49.54% to 51.09%), consistent with the expected GC composition of the bovine transcriptome. The average error rate was 0.01% across all libraries, confirming the accuracy of the sequencing platform. Collectively, these results indicate that the RNA-seq dataset is of high quality and suitable for subsequent differential gene expression and functional enrichment analyses.

### 3.4. Gene Differential Expression Analysis

**Figure 4:**
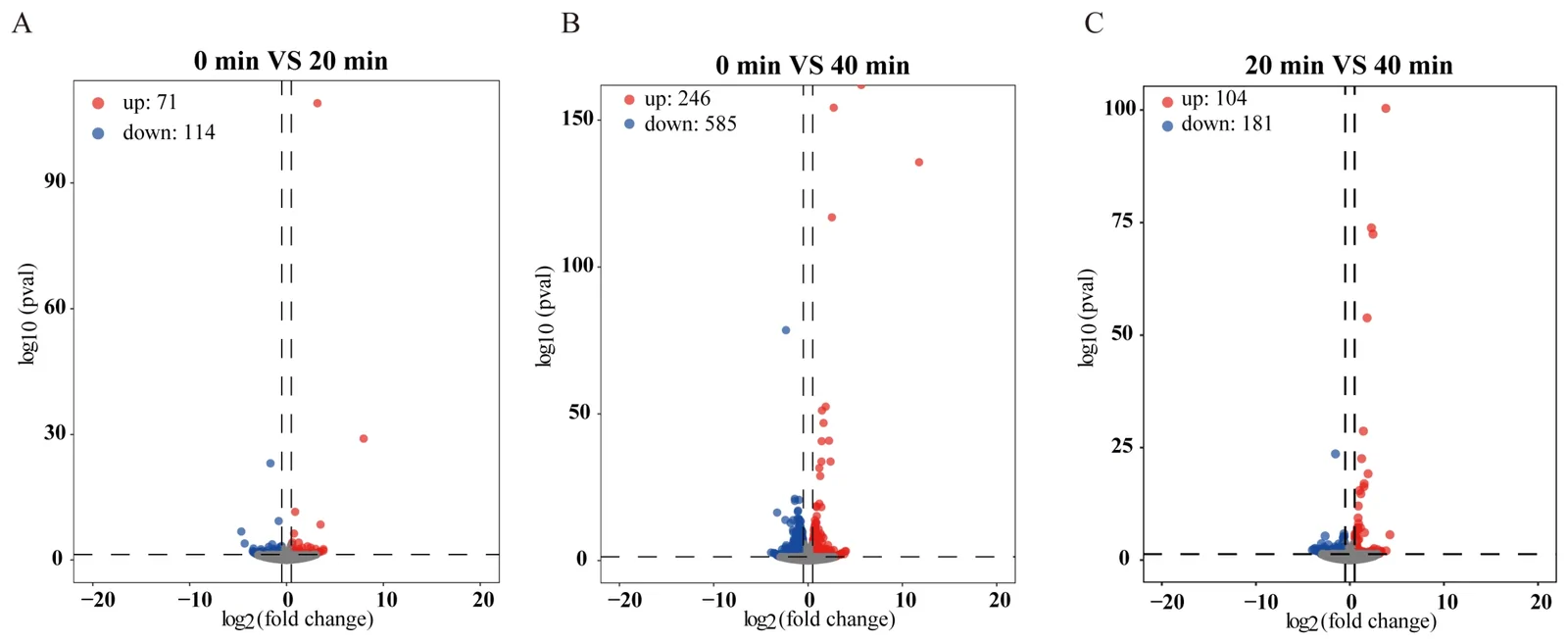
Volcano plot of differentially expressed genes. (**A**) 0 min vs. 20 min; (**B**) 0 min vs. 40 min; (**C**) 20 min vs. 40 min. Red dots indicate significantly upregulated genes, blue dots indicate significantly downregulated genes, and gray dots indicate genes with no significant difference.

To comprehensively characterize the transcriptional responses of bovine granulosa cells to acute heat stress, we performed RNA-seq analysis on cells exposed to 43 °C for 0 min (control), 20 min, and 40 min. A total of 185 DEGs were identified in the early response (0 min vs. 20 min), of which 71 genes (38.4%) were significantly upregulated, and 114 genes (61.6%) were downregulated (Figure 4A). Among the upregulated genes, the most prominent were classical heat shock proteins, including HSPA1A, HSPA6, and DNAJB1, confirming the rapid activation of the cytoprotective chaperone response. Additionally, immediate-early transcription factors such as *FOS*, *JUN*, and *EGR1* were significantly upregulated, suggesting a coordinated transcriptional cascade initiated within 20 min of thermal insult. Notably, several metabolism-related genes were among the downregulated DEGs, including *FBP2*, *HAO2*, and *ALDOB*, indicating an early suppression of metabolic pathways. In addition, genes involved in ciliogenesis, such as *CFAP45*, *CFAP68*, and *DNAAF1*, were significantly downregulated at 20 min, suggesting that heat stress may impair primary cilium assembly even at the early stage.

A substantially larger transcriptional response was observed at 40 min in the prolonged response (0 min vs. 40 min), with 831 DEGs identified, comprising 246 upregulated genes (29.6%) and 585 downregulated genes (70.4%) (Figure 4B). This 4.5-fold increase in DEG numbers compared to the 20-min time point demonstrates a profound time-amplification effect of heat stress on the granulosa cell transcriptome. The upregulated gene set at 40 min included not only sustained heat shock genes (*HSPA1A*, *HSPA6*, *HSPB1*, *DNAJB1*, *DNAJB4*, *CRYAB*) but also a broad spectrum of stress-responsive transcription factors (*ATF3*, *DDIT3*, *NFKBIZ*), pro-apoptotic regulators (*BBC3*, *TRIB2*, *RIPK4*), and inflammatory mediators (*IL1B*, *TNFAIP3*, *CCL27*). Conversely, the downregulated genes at 40 min were extensively enriched in functional categories related to steroidogenesis (*CYP1B1*, *CYP2W1*, *HSD17B14*), gluconeogenesis (*FBP2*, *HAO2*, *ALDOB*), and cilium assembly (*CFAP45*, *CFAP68*, *DNAAF1*, *DNAAF3*, *DNAAF4*, *DNAH12*, *DNAI1*), indicating a broad suppression of granulosa cell-specific differentiated functions.

To capture the transcriptional dynamics occurring between the two heat exposure durations, we further analyzed DEGs between the 20 min and 40 min groups. This comparison identified 285 DEGs, with 104 genes (36.5%) upregulated and 181 genes (63.5%) downregulated (Figure 4C). The upregulated genes in this transitional phase included late-induced heat shock proteins (*DNAJA4*, *HSPH1*, *BAG3*), apoptosis-related genes (*DDIT3*, *GADD45G*), and inflammatory regulators (*NFKBIZ*, *ZFP36L1*). The downregulated genes were predominantly associated with mitochondrial function (*NDUFA4L2*), steroid metabolism (*CYP2W1*), and ciliary components (*CFAP68*, *DNAAF3*), reflecting the progressive deterioration of cellular homeostasis as heat stress persists.

Overall, these results demonstrate a striking time-dependent amplification of the heat stress-induced transcriptomic response in bovine granulosa cells. The number of DEGs expanded from 185 at 20 min to 831 at 40 min, with 285 genes commonly dysregulated across both time points representing the core heat stress signature. The progressive shift from a focused cytoprotective transcriptional program at 20 min to a broad pathological reprogramming involving apoptosis, inflammation, and metabolic suppression at 40 min underscores the dynamic nature of the cellular response to sustained hyperthermic challenge.

### 3.5. Functional Enrichment Analysis of Time-Dependent DEGs

To gain mechanistic insights into the transcriptional reprogramming induced by acute heat stress, we performed Gene Ontology (GO) and Kyoto Encyclopedia of Genes and Genomes (KEGG) pathway enrichment analyses on the DEGs from three pairwise comparisons: 0 vs. 20 min, 0 vs. 40 min, and 20 vs. 40 min.

**Figure 5:**
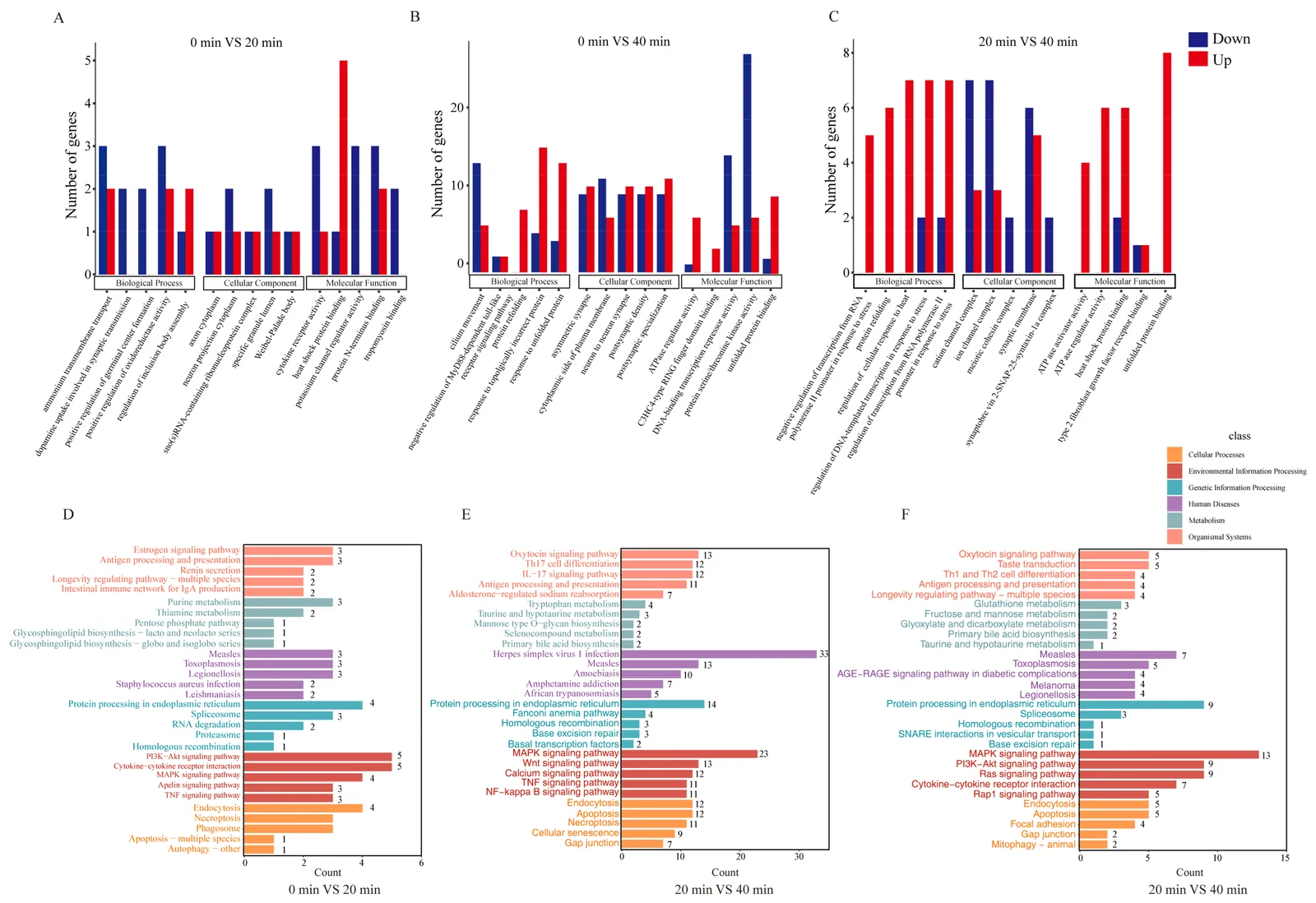
Functional enrichment analysis of DEGs at different time points following heat stress. (**A**–**C**) Gene Ontology (GO) enrichment analysis of DEGs identified in the 0 vs. 20 min (**A**), 0 vs. 40 min (**B**), and 20 vs. 40 min (**C**) comparisons. (**D**–**F**) KEGG pathway enrichment analysis of DEGs in the corresponding pairwise comparisons (**D**–**F**). Only significantly enriched terms and pathways are shown (adjusted *p* < 0.05).

For the early response (0 min vs. 20 min), GO analysis demonstrated that the 185 DEGs were significantly enriched in biological processes including ammonium transmembrane transport, positive regulation of germinal center formation, and cellular components such as axon cytoplasm (Figure 5A). KEGG pathway analysis further associated these DEGs with the estrogen signaling pathway, RNA degradation pathway, PI3K-AKT signaling pathway, and MAPK signaling pathway (Figure 5D), indicating that the early heat shock response engages both hormone signaling cascades and stress-activated kinase networks.

In the late response (0 min vs. 40 min), GO enrichment revealed a marked shift toward protein homeostasis-related terms, including the cytoplasmic side of the plasma membrane, protein refolding, and response to unfolded protein (Figure 5B), consistent with the massive induction of heat shock proteins observed at this time point. KEGG analysis identified the MAPK signaling pathway, WNT signaling pathway, and TNF signaling pathway as the predominant enriched pathways (Figure 5E), suggesting that prolonged heat exposure triggers inflammatory and developmental signaling disruptions beyond the canonical heat shock response.

To characterize the transcriptional dynamics occurring between the two heat treatment durations, we additionally analyzed the DEGs between the 20 min and 40 min groups. These genes were predominantly enriched in GO terms associated with regulation of cellular response to heat stress, DNA-templated transcription, ion channel complexes, and meiotic cohesion complexes (Figure 5C). KEGG analysis further pinpointed the PI3K-AKT signaling pathway, MAPK signaling pathway, and Ras signaling pathway as the major enriched pathways (Figure 5F), implying that the transition from early to late heat stress involves progressive activation of cell survival and proliferation-related signaling axes.

**Figure 6:**
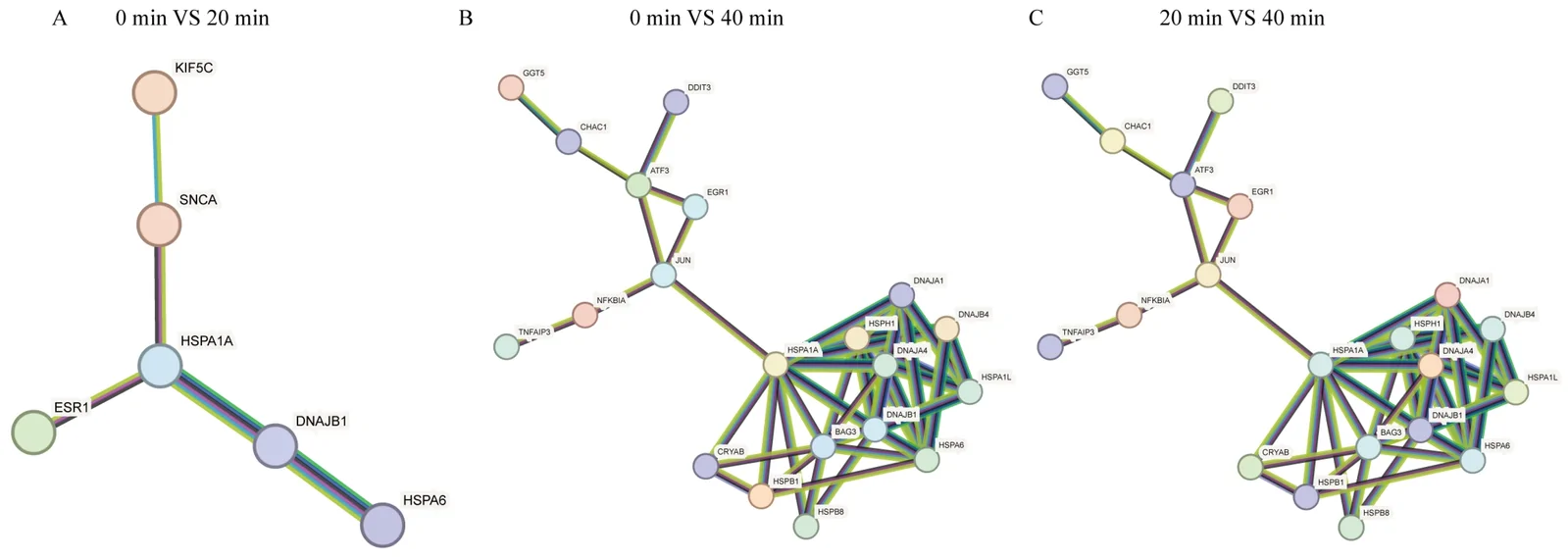
Protein–protein interaction (PPI) network of heat stress-responsive DEGs. (**A**) 0 min vs. 20 min; (**B**) 0 min vs. 40 min; (**C**) 20 min vs. 40 min. The network was constructed using the STRING database. Nodes represent proteins, and edges indicate predicted functional associations. The size of each node reflects the degree of connectivity, with larger nodes indicating higher centrality. *HSPA1A*, *EGR1*, and *JUN* are highlighted as potential hub genes. Edge colors indicate interaction evidence types: light blue, curated databases; pink, experimentally determined; green, gene neighborhood; red, gene fusions; dark blue, gene co-occurrence; yellow, text mining; black, co-expression.

Furthermore, a protein–protein interaction (PPI) network was constructed using the STRING database to predict functional associations among the DEG-encoded proteins. As shown in Figure 6, the network exhibited a highly interconnected topology, with *HSPA1A*, *EGR1*, and *JUN* emerging as central hub nodes. This suggests that these three genes may serve as key molecular regulators orchestrating the cellular response to heat stress in bovine granulosa cells.

### 3.6. Confirmation of RNA-Seq Experiment

**Figure 7:**
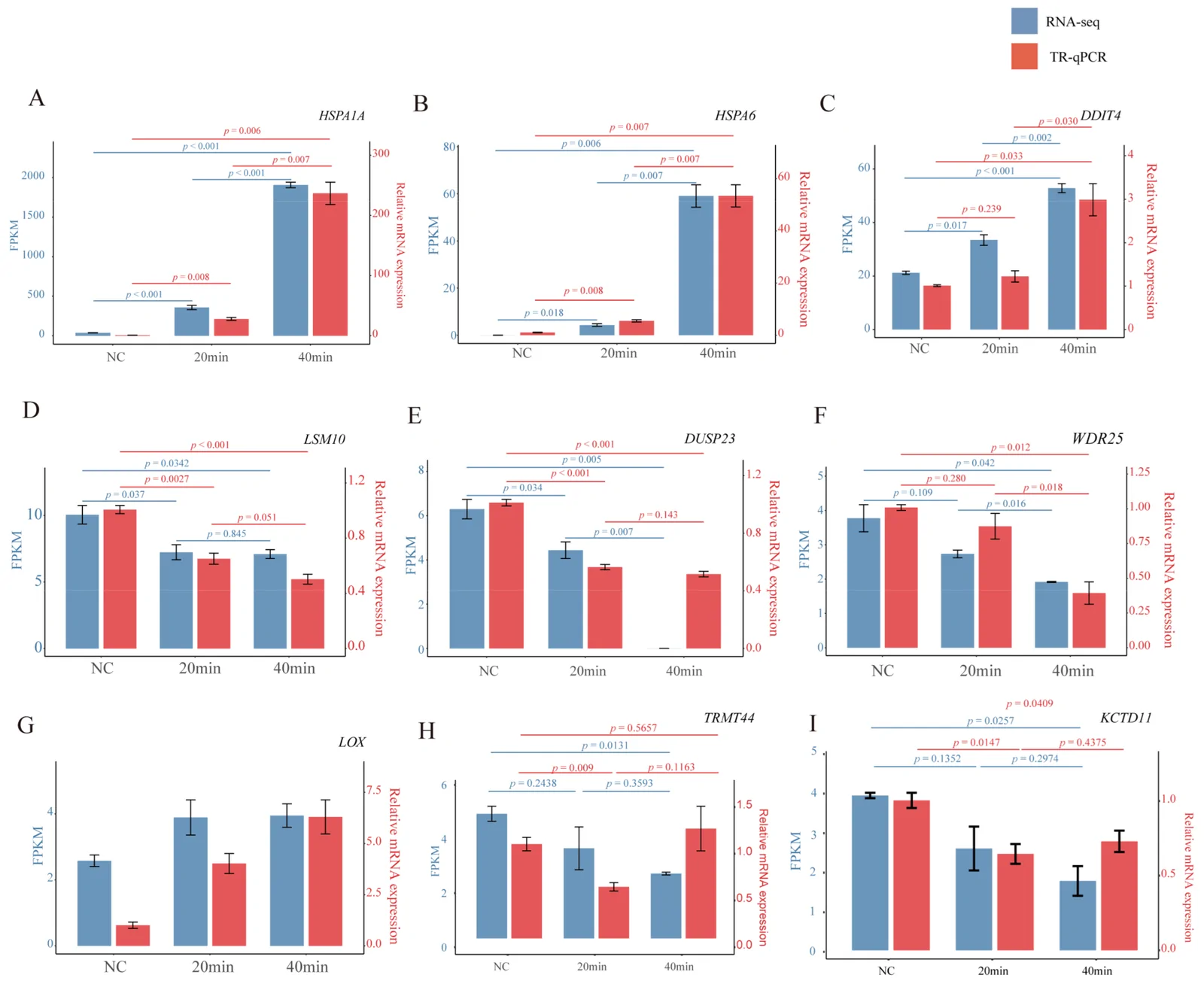
Relative expression levels of DEGs by FPKM and RT-qPCR in different treatments. (**A**) *HSPA1A*. (**B**) *HSPA6*. (**C**) *DDIT4*. (**D**) *LSM10*. (**E**) *DUSP23*. (**F**) *WDR25*. (**G**) *LOX*. (**H**) *TRMT44*. (**I**) *KCTD11*. FPKM values of RNA-seq (blue) are on the left y-axis, and the relative expression level by RT-qPCR (red) is on the right y-axis.

Nine protein-coding DEGs, namely *HSPA1A*, *HSPA6*, *DDIT4*, *LSM10*, *DUSP23*, *WDR25*, *LOX*, *TRMT44*, *KCTD11*, were selected for qPCR validation. These genes were chosen to represent a range of fold-change magnitudes and functional categories (e.g., heat shock response, stress signaling, transcriptional regulation, and extracellular matrix remodeling) based on the RNA-seq results. The results demonstrated a high degree of consistency between RNA-Seq and the qPCR data (Figure 7), confirming the reliability of the relative gene expression measurements obtained in the current RNA-Seq dataset.

## 4. Discussion

Heat stress is a major environmental challenge that compromises reproductive performance in dairy cattle, primarily through deleterious effects on ovarian follicular cells. Granulosa cells (GCs), which provide essential nutritional and hormonal support for oocyte maturation, are particularly vulnerable to hyperthermic insult [1,2,15]. In our previous study, we demonstrated that heat stress (43 °C for 20 min) induced oxidative stress, apoptosis, and mitochondrial dysfunction in bovine GCs. Building upon these findings, the present study systematically investigated the temporal dynamics of mitochondrial morphological alterations and transcriptomic reprogramming in bovine GCs subjected to acute heat stress for 20 and 40 min. Our results reveal that acute heat stress induces a time-dependent amplification of transcriptomic responses, accompanied by progressive mitochondrial structural injury and calcium dyshomeostasis, providing a mechanistic framework for understanding early heat stress-induced ovarian dysfunction.

The heat shock response represents the first line of cellular defense against proteotoxic stress. Our data reveal that *HSPA1A* expression was significantly upregulated as early as 20 min of heat exposure, whereas *HSP90* did not reach statistical significance until 30 min. This differential activation threshold suggests that *HSPA1A*, as the major inducible heat shock protein, is preferentially and rapidly mobilized to counteract protein misfolding, while the constitutively expressed *HSP90* requires prolonged stimulation for transcriptional engagement. This finding is consistent with the classical paradigm that *HSF1* rapidly activates *HSPA1A* transcription within minutes of thermal insult, whereas *HSP90*, as part of a negative feedback loop with *HSF1*, exhibits a delayed response. Notably, although *HSPA1A* was significantly upregulated at 20 min, the full engagement of the broader chaperone network—including *HSP90*, *DNAJB1*, *HSPB1*, and *CRYAB*—was not achieved until 40 min, reflecting a hierarchical activation of the heat shock machinery.

Catherine et al. reported that knockdown of *HSPA1A* expression contributes to the protection of differentiated human neuronal cells from heat stress, suggesting a context-dependent role of *HSPA1A* in cell survival [16]. Similarly, Wen et al. demonstrated that colony-stimulating factor 2 (*CSF2*) inhibits *HSPA1A* expression to facilitate yak blastocyst formation and increase cell numbers [17]. These observations, together with our finding that *HSPA1A* is dramatically upregulated in heat-stressed GCs, highlight the dual nature of *HSPA1A* function—while its induction is essential for protein homeostasis and cytoprotection, sustained overexpression may also predispose cells to inflammatory or apoptotic signals under prolonged stress conditions. This duality warrants further investigation in the context of ovarian follicle development.

Mitochondria serve as the central hub for steroid hormone biosynthesis in GCs, and their functional integrity is critical for maintaining hormone-secreting capacity [18]. Our results demonstrated that intracellular Ca^2+^ concentration exhibited a biphasic pattern—markedly elevated at 20 min but significantly reduced at 40 min—accompanied by progressive mitochondrial swelling and cristae disruption at both time points, with more severe damage at 40 min. This “rise-then-fall” calcium dynamic carries substantial pathophysiological significance. The early (20 min) Ca^2+^ elevation likely originates from heat stress-induced endoplasmic reticulum (ER) stress: accumulation of misfolded proteins within the ER lumen activates PERK and IRE1 pathways, which promote ER calcium release through IP3 receptors [19]. Moderate cytosolic Ca^2+^ elevation can transiently enhance mitochondrial oxidative metabolism to meet increased energy demands. However, sustained calcium overload drives massive Ca^2+^ influx into the mitochondrial matrix, triggering the opening of the mitochondrial permeability transition pore (mPTP), leading to mitochondrial swelling, cristae disintegration, and outer membrane rupture—findings that perfectly align with our TEM observations [20]. The significant decline in cytosolic Ca^2+^ at 40 min likely reflects depletion of ER calcium stores and functional decompensation of calcium pumps (PMCA and SERCA) against a backdrop of ATP depletion.

Meng et al. reported that LPS induced mitochondrial dysfunction and ER stress by upregulating ER stress-related genes and proteins in bovine mammary epithelial cells [21]. Hu et al. demonstrated that a decrease in oxidative phosphorylation (OXPHOS) and mitochondrial disequilibrium are involved in large white follicle development in hens [22]. Liang et al. reported that nicotinamide mononucleotide (NMN) supplementation improved energy metabolism and mitochondrial density and morphology, thereby increasing ovarian reserve and health status in mouse GCs [23]. Our study extends these findings by demonstrating that heat stress induces mitochondrial dysfunction within 20 min, with progressive deterioration at 40 min. We propose that mitochondrial swelling-induced uncoupling of OXPHOS and reduced ATP synthesis further aggravates the energy crisis of calcium pumps, forming a vicious cycle of “calcium dysregulation → mitochondrial damage → ATP insufficiency → calcium pump failure → exacerbated calcium dysregulation,” ultimately driving GCs toward an irreversible injury trajectory. Importantly, the fact that mitochondrial structural damage occurs within 20 min—even before the full activation of the heat shock protein network—suggests that mitochondria are among the earliest cellular targets of heat stress, and that early intervention at this stage may be critical for preserving GC function.

Our transcriptomic analysis revealed a striking time-dependent amplification of the heat stress response, with DEGs expanding from 185 at 20 min to 831 at 40 min, and 285 genes commonly dysregulated across both time points representing the core heat stress signature. This nonlinear expansion underscores the transition from a focused cytoprotective program to a broad pathological reprogramming. The 20-min transcriptome was dominated by classical heat shock proteins (HSPA1A, HSPA6, DNAJB1) and immediate-early transcription factors (*FOS*, *JUN*, *EGR1*). KEGG pathway analysis further revealed enrichment of the estrogen signaling pathway, PI3K-AKT signaling pathway, and MAPK signaling pathway. The PI3K/AKT pathway plays a crucial role in oocyte maturation, with AKT specifically regulating mitosis, meiosis, and early embryo development. Hui et al. reported that progesterone coordinates with follicle-stimulating hormone (FSH) to regulate follicle growth via the PI3K/AKT and MAPK signaling pathways in mice [24]. Soek et al. demonstrated that canine oviductal exosomes improve oocyte development via the EGFR/MAPK signaling pathway [25]. Our observation that heat stress for 20 min significantly increased *HSPA1A* expression and disrupted PI3K/AKT pathway components suggests that even at this early time point, GCs are experiencing metabolic and signaling disturbances that may compromise their supportive capacity for oocytes.

Among the 185 DEGs, PPI network analysis identified *HSPA1A*, *ESR1*, *EDN1*, *FGF18*, *KIF5C*, and *RET* as hub genes. Rui et al. suggested that estrogen may regulate goat oocyte meiosis arrest by decreasing the number of transzonal projections (TZPs) via estrogen-mediated *GPER* activation during follicle development [26]. In our study, heat stress for 20 min significantly decreased *ESR1* expression while increasing *HSPA1A* expression, suggesting a trade-off between the heat shock response and estrogen signaling. The downregulation of *ESR1* may impair the ability of GCs to respond to estrogenic signals, potentially contributing to follicular growth arrest. Currently, there is limited research on the expression of *EDN1*, *FGF18*, and *KIF5C* in relation to follicle development, indicating a need for further investigation.

At 40 min, the transcriptional landscape expanded substantially to encompass apoptosis (*DDIT3*, *BBC3*, *TRIB2*, *RIPK4*), inflammation (*IL1B*, *TNFAIP3*, *NFKBIZ*, *CCL27*), steroidogenic suppression (*CYP1B1*, *CYP2W1*, *HSD17B14*), and ciliopathy (*CFAP45*, *CFAP68*, *DNAAF1*, *DNAAF3*). KEGG analysis further identified enrichment of the WNT signaling pathway, TNF signaling pathway, and MAPK signaling pathway. Feng et al. reported that WNT plays a vital role in various cellular and physiological processes through different signaling pathways during embryogenesis [27]. Xing et al. demonstrated that *SMAD4* activates the WNT signaling pathway to inhibit GC apoptosis [28]. Our results show that prolonged heat stress may disrupt WNT signaling, potentially removing a survival signal that normally protects GCs from apoptosis.

The downregulation of ciliogenesis genes (*CFAP45*, *CFAP68*, *DNAAF1*, *DNAAF3*, *DNAAF4*, *DNAH12*, *DNAI1*) at 40 min is particularly noteworthy. Primary cilia function as cellular sensory organelles that modulate hedgehog signaling and mechanotransduction in GCs. Their transcriptional suppression implies that prolonged heat stress may impair the ability of GCs to perceive and transduce extracellular signals, including FSH, thereby compromising oocyte maturation and ovulation [29]. This finding reveals a previously unrecognized target of heat stress in GCs and highlights the importance of ciliary integrity for normal ovarian function.

The 285 genes commonly dysregulated across both time points constitute a robust core heat stress signature that is independent of exposure duration. This signature is enriched in heat shock proteins (*HSPA1A*, *HSPA6*, *HSPB1*, *CRYAB*, *DNAJB1*), stress-responsive transcription factors (*ATF3*, *EGR1*, *JUN*, *FOS*), metabolic regulators (*DDIT4*, *INSIG1*, *GADD45G*), and numerous long non-coding RNAs. We propose that this core gene set could serve as a molecular indicator panel for evaluating the extent of heat stress injury in GCs. Among these, the *HSPA1A*/*DDIT3* expression ratio may be particularly informative—*HSPA1A* represents the cellular protein folding buffering capacity, whereas *DDIT3* (CHOP) is a key executor of ER stress-induced apoptosis. Their combined ratio could reflect the balance between survival buffering and death commitment. This further supports the notion that *HSPA1A* serves as a critical node linking heat shock response with steroidogenic function. The abundant lncRNAs within the core signature suggest that non-coding RNAs may play underappreciated roles in the heat stress response, acting as competing endogenous RNAs (ceRNAs) to regulate miRNA availability, or as scaffolds guiding chromatin remodeling complexes to heat shock gene promoters [30]. Future studies should investigate the functional significance of these lncRNAs in the context of heat stress.

Based on our multidimensional evidence, we propose a “mitochondria–calcium–transcriptome” axis as an integrated framework for understanding heat stress-induced GC dysfunction. In this model, heat stress first acts on the plasma membrane and ER, causing early calcium influx and ER calcium release. Calcium overload drives mitochondrial calcium uptake, triggering mPTP opening and mitochondrial swelling (confirmed by TEM observation), with uncoupling of OXPHOS resulting in decreased ATP synthesis. Concurrently, the accumulation of misfolded proteins in the ER lumen triggers the UPR, further exacerbating injury through calcium signaling and apoptotic pathways. At the transcriptional level, the early (20 min) protective program (HSPs, antioxidant genes) is followed by the late (40 min) pathological shift toward pro-apoptotic, pro-inflammatory, and metabolic suppression signatures, forming a positive feedback loop with mitochondrial functional failure. Ultimately, GC steroidogenic capacity is severely compromised, ciliary function is impaired, and an inflammatory microenvironment is established, collectively leading to follicular developmental impairment and reduced fertility. The 20-to-40-min transition revealed by our transcriptomic data identifies a critical intervention window—the period during which cytoprotective mechanisms are still active but pathological pathways have begun to emerge. Interventions aimed at reinforcing the early HSP response or mitigating mitochondrial calcium overload during this window may offer therapeutic potential for preserving GC function and female fertility under hyperthermic conditions.

Several limitations of this study should be acknowledged. Transcriptomic changes need further validation at the protein and metabolite levels, as mRNA abundance does not always reflect protein activity and metabolic flux. Future studies should integrate proteomics and metabolomics to confirm the functional consequences of the transcriptional reprogramming we identified. Finally, in vivo heat stress models (e.g., housing dairy cows under high-temperature and high-humidity conditions) are needed to validate the clinical relevance of our core signature and hub genes (*HSPA1A*, *ESR1*, *JUN*) as potential biomarkers for fertility assessment.

## 5. Conclusions

This study demonstrates that acute heat stress induces time-dependent mitochondrial injury and transcriptomic reprogramming in bovine granulosa cells. Calcium exhibited a biphasic pattern—elevated at 20 min but depleted at 40 min—concurrent with progressive mitochondrial swelling and cristae disruption. Transcriptomic profiling identified 185 DEGs at 20 min and 831 at 40 min, with 285 genes forming a core signature. Functional enrichment revealed a shift from cytoprotection at 20 min to pathological reprogramming involving apoptosis, inflammation, and steroidogenic suppression at 40 min. PPI analysis highlighted *HSPA1A*, *ESR1*, and *JUN* as hub genes. We propose a “mitochondria–calcium–transcriptome” axis model wherein calcium dyshomeostasis drives mitochondrial damage, synergizing with transcriptional reprogramming to cause granulosa cell dysfunction. The 20-to-40-min transition reveals a critical intervention window. Our findings provide a mechanistic framework for early heat stress-induced ovarian impairment and identify molecular targets for preserving fertility in dairy cattle.

## Figures and Tables

**Table 1:** Primers for q RT–PCR.

Gene	Primer Sequence (5′-3′)	bp
*HSP90*	F: CTGGAAGGAGACGACGACAC	104
	R: ACACACTGGAGGGAATGGAG	
*HSPA1A*	F: GGACCTGCTGTTGCTGGAC	102
	R: TTCGTGGGGATGGTGGAGTT	
*GAPDH*	F: AAGTTCAACGGCACAGTCA	365
	R: GTCATAAGTCCCTCCACGAT	
*HSPA6*	F:GAAACCACAACCATGTCCGC	475
	R: AGTCGTTGAAGTAGGCAGGC	
*DDIT4*	F:AACTCCCACCCCAAATCAGC	163
	R: CACCCCATCCAGGTATGCAG	
*LSM10*	F:AGCAAGCGTGGAAGAATGGA	153
	R:AACGTTGTCTATGCGTCCGT	
*DUSP23*	F:GTGTACGGCATCTGGTGTCA	416
	R:GTCTCTGGCCCCATCTAACG	
*WDR25*	F:TTCCAGCACCGACGGAAC	386
	R:GCCATCGTAGTCTCTGGCTG	
*LOX*	F:CACATCGTGTGACTACGGCT	295
	R:TTGGGAGTTTTGGCTTGCTT	
*TRMT44*	F:TGGTTCTTCGCTCATCACCC	285
	R:TGCTGTAAAGACGCAGAGCA	
*KCTD11*	F:TTTCCGTCTGGAATGGGCTC	236
	R:AGTGCCGAACAAAGCGTAGA	

**Table 2:** Raw data statistical analysis.

Sample	Raw Data Reads	Clean Data Reads	Raw Data Reads	Clean Data Base	Q20/%	Q30/%	GC/%	Error/%
0 min-1	47,156,742	46,145,500	7,073,511,300	6,902,987,672	98.67	96.03	50.92	0.01
0 min-2	45,328,898	44,380,338	6,799,334,700	6,642,510,466	98.73	96.21	50.25	0.01
0 min-3	50,321,332	49,168,518	7,548,199,800	7,348,485,152	98.67	96.06	51.08	0.01
20 min-1	45,924,812	44,473,260	6,815,971,800	6,652,182,646	98.69	96.07	49.54	0.01
20 min-2	45,924,082	44,599,500	6,888,612,300	6,668,339,408	98.64	95.95	51.09	0.01
20 min-3	45,796,108	44,774,134	6,869,416,200	6,692,386,802	98.69	96.10	50.69	0.01
40 min-1	49,104,496	47,964,404	7,365,674,400	7,169,989,062	98.67	96.05	50.70	0.01
40 min-2	42,983,198	41,944,536	6,447,479,700	6,269,956,812	98.66	96.00	50.77	0.01
40 min-3	46,192,426	45,186,884	6,928,863,900	6,765,127,522	98.69	96.10	50.05	0.01

## Data Availability

The data presented in this study are available on request from the corresponding author. The data are not publicly available due to institutional privacy policies.
